# Changes in work tasks and organization of general practice in Norway during the COVID-19 pandemic: results from a comparative international study

**DOI:** 10.1186/s12875-023-02146-x

**Published:** 2023-10-28

**Authors:** Torunn Bjerve Eide, Esther van Poel, Sara Willems, Frode F. Jacobsen

**Affiliations:** 1 Department of General Practice, Institute of Health and Society, University of Oslo, Oslo, Norway; 2 Department of Public Health and Primary Care, Ghent University, Ghent, Belgium; 3 Quality and Safety Ghent, Department of Public Health and Primary Care, Ghent University, Ghent, Belgium; 4 Centre for Care Research West, Western Norway University of Applied Sciences, Bergen, Norway; 5 VID Specialized University, Bergen, Norway

**Keywords:** Primary health care, General practice, Norway, COVID-19, PRICOV-19, Quality of care, Infection prevention and control, Patient flow management, Primary care workforce

## Abstract

**Background:**

The COVID-19 pandemic led to huge and rapid changes in general practice in Norway as in the rest of Europe. This paper aims to explore to what extent the COVID-19 pandemic changed the work tasks and organization of Norwegian general practice.

**Material and method:**

We analysed data from the Norwegian part of the international, cross-sectional PRICOV-19 study, collecting data from general practice via an online self-reported questionnaire. We included 130 Norwegian general practices, representing an estimated 520 Norwegian general practitioners (GPs). All Norwegian GPs were invited to participate. In the analyses, we focused on items related to the use of alternatives to face-to-face consultations, changes in the workload, tasks and delegated responsibilities of both the GPs and other personnel in the GP offices, adaptations in routines related to hygiene measures, triage of patients, and how the official rules and recommendations affected the practices.

**Results:**

There was a large and significant increase in the use of all forms of alternative consultation forms (digital text-based, video- and telephone consultations). The use of several different infection prevention measures were significantly increased, and the provision of hand sanitizer to patients increased from 29.6% pre-pandemic to 95.1% since the pandemic. More than half of the GPs (59.5%) reported that their responsibilities in the practice had increased, and 41% were happy with the task shift. 27% felt that they received adequate support from the government; however, 20% reported that guidelines from the government posed a threat to the well-being of the practice staff. We found no associations with the rurality of the practice location or size of the municipalities.

**Conclusion:**

Norwegian GPs adapted well to the need for increased use of alternatives to face-to-face consultations, and reported a high acceptance of their increased responsibilities. However, only one in four received adequate support from the government, which is an important learning point for similar situations in the future.

## Background

The first Norwegian COVID-19 case was confirmed 26th February 2020, signalling the beginning of the pandemic. The Norwegian government launched the most intrusive population restrictions ever given in peacetime in Norway [1]. Although less intrusive in Norway than in most high-income countries, Norwegian infection control measures have been reported as relatively efficient in international comparisons [2, 3]. During the period covered by the research presented in this article, Norway had one of the world’s lowest COVID-19-related mortality rates [1]. In line with the bulk of high-income countries [4], most Norwegian patients were treated in the municipalities rather than in the specialised health services [1]. General practitioners (GPs) form a cornerstone of Norwegian healthcare services, and all Norwegian inhabitants are assigned their own regular GP. Most Norwegian GPs are self-employed and work on a combination of capitation and fee-for-service [5]. The government reimbursed all patient contacts regarding suspected or confirmed COVID-19 infection with no patient fee.

Norwegian GPs were asked to identify which of their patients were particularly vulnerable to health risks related to infection, in order to prioritize them for vaccination. The government enabled a digitalization tool to make this selection possible. The GPs were also responsible for offering their patient population the opportunity to be vaccinated against the SARS-CoV-2 virus. The municipal health services were assigned the task of clinical follow-up of patients with symptoms of airway infections and those who had confirmed COVID-19 infection. The implementation of this task varied among the 356 municipalities, as each municipality was free to decide on a suitable organisation in their particular circumstances. The organisation of this work developed throughout the pandemic. Each GP practice initially had to prepare for the possible reception of COVID-19 infected patients. However, as the pandemic evolved, a highly organised reception and evaluation of patients with airway infection symptoms was launched. In smaller communities, this was often handled by the GP practices, but in larger cities, the patients with symptoms of infections were mainly handled by designated respiratory clinics [2].

This paper aims to explore to what extent the COVID-19 pandemic changed the work tasks and the organisation of general practice in Norway, with a comparison to an international cohort.

## Methods

### Study design and setting

The material in this study stems from the PRICOV-19 study, an international study under the coordination of Ghent University (Belgium) [6]. The Norwegian results from this study are compared to results from the total PRICOV-19 cohort. This multi-country cross-sectional study aims to explore how primary care practices were organised during the COVID-19 pandemic to guarantee high-quality care; how the task roles changed and how the pandemic affected the wellbeing of care providers; and whether differences could be found between types of practices and/or healthcare systems. Data were collected in 37 European countries and Israel. The published study protocol describes the project in more detail [6].

### Measurements

Data were collected via an online self-reported questionnaire. The questionnaire was developed at Ghent University in multiple phases, including a pilot study among 159 GP practices in Flanders (Belgium). More details are described elsewhere [6]. The questionnaire consists of 53 items divided into six domains: (a) infection prevention; (b) patient flow for COVID- and non-COVID care; (c) dealing with new knowledge and protocols; (d) communication with patients; (e) collaboration and wellbeing of the respondent; (f) and characteristics of the respondent and practice. The questionnaire was translated into 38 languages following a standard procedure. The Research Electronic Data Capture (REDCap) platform was used to host the questionnaire in all languages, send out invitations to the national samples of GP practices, and securely store the answers from the participants [7].

### Sampling and recruitment

The Norwegian data were collected between March and May 2021. All Norwegian GPs (at the time, 5135 GPs in 1368 GP practices) were invited to participate via the digital information letter from the Norwegian Association of General Practice, which was sent to all GPs about twice per week in this phase of the pandemic. Respondents were also invited via a closed Facebook group for Norwegian GPs, which had about 4800 members at the time. The invitation and introduction highlighted that only one GP per practice should answer the questionnaire. We aimed to sample 200 Norwegian GP practices. One hundred ninety-one questionnaires were answered, but a relatively large proportion was incomplete. In this paper, we excluded cases where only the demographic information was completed. This resulted in a sample of 130 questionnaires for analysis of the Norwegian data.

### Variables of interest

We focused on items related to change in the use of alternative/electronic consultation forms (three items), changes in the workload, tasks, and delegated responsibilities of both the GPs and other personnel in the GP offices (7 items), change in routines related to hygiene measures (8 items), and triage of patients (2 items). We also looked at how the official rules and recommendations affected the GP practices (3 items). For detailed information on the included items, see Tables 2, 3, 4 and 5.

### Data analysis

Ghent University was responsible for cleaning the data and supplying the results for the international cohort. The Norwegian team was responsible for the data analyses for this paper. Demographic variables are reported using descriptive statistics. All reported percentages are valid, with missing answers excluded. For some variables, the participants were asked to indicate whether they agree, strongly agree, were neutral, disagreed or strongly disagreed. For these, we grouped the neutrals with the missing data, and analysed possible differences between *agree + strongly agree* versus *disagree + strongly disagree*. We used Fishers Exact Test to determine associations between variables. The criterion of statistical significance (two-sided, p) was determined at 0.05. The analyses were performed using IBM SPSS Statistics 27 (IBM Corp., Armonk, N.Y., USA).

### Ethics approval

The study was conducted according to the guidelines of the Declaration of Helsinki. The Research Ethics Committee of Ghent University Hospital approved the protocol of the PRICOV-19 study (BC-07617). No identifiable data were collected, and all participants gave informed consent on the first page of the online questionnaire. Therefore, the study was not eligible for approval by the Norwegian Regional Ethical research Committee (document ref. 231984, January 2021).

## Results

### Description of the sample

In the analyses, data from 130 Norwegian GPs were included. Of the respondents, 126 were regular GPs, two were GP locums, and two missed information regarding their position in the practice. Further characteristics are found in Table 1. The respondents were asked to answer on behalf of their practice. The median number of GPs per practice was four. Hence, the answers represent an estimate of 520 GPs, about 10 percent of Norway’s GP population. The practices represented both small and large municipalities, urban and rural settings, and all four Norwegian Health Regions. In the Total PRICOV-19 study, 5961 respondents from 29 countries completed the questionnaire.

**Table 1:** Demographic information of the participating GP practices in Norway (*n*=130)

**Characteristic of the practice**	**N (%)**
Specialist in General Practice?^a^	103 (81.1)
Number of inhabitants in your municipality^b^	
Less than 10 0000	23 (18)
10 000-100 000	72 (56.3)
More than 100 000	33 (25.8)
Practice location	
Big (inner)city	33 (25.4
Suburbs	15 (11.5)
(Small) town	43 (33.1)
Mixed urban-rural	11 (8.5)
Rural	28 (21.5)
Missing	0
Health region of the practice	
Northern Norway	20 (15.4)
Central Norway	13 (10)
Western Norway	22 (16.9)
South-Eastern Norway	75 (57.7)
Number of regular GPs in practice^b^	
Range	1-12
Mean	4.4
Median	4
Number of patients on your list^b^	
Range	300-2050
Mean	1065
Median	1078
Number of patients registered to the practice	
Range	500-13500
Mean	4877.5
Median	4500

*GP* General practitioner

Missing: ^a^3, ^b^2

### Available time for professional update

Seventeen (13.9%) of respondents agreed or strongly agreed that before the pandemic they had enough protected time for reading guidelines and literature. This increased to 24 (19.5%) after the start of the pandemic. The difference was not significant. In the complete international data from the PRICOV-19 study, as many as 24.2% indicated enough protected time for professional update, and no change was observed after the pandemic. The number of Norwegian practices that had daily or weekly meetings to discuss new directives increased from 45 (36.8%) to 72 (58.5%, *p*<0.01) after the start of the pandemic. Details are found in Table 2.

**Table 2:** Change in time used for professional update

	**Before COVID-19**^a^ **N (%)**	**Since COVID-19**^a^ **N (%)**	***P* value**	**Total Pricov-19 cohort** **N (%)**	
				Before COVID-19	Since COVID-19
Frequency of team meetings to discuss existing, new or amended directives					
Never	11 (9)	5 (4.1)	0.12	690 (14.5)	659 (13.9)
Weekly or less	109 (89.3)	101 (82.1)	0.21	3448 (74.8)	2926 (61.6)
Daily	2 (1.6)	15 (12.2)	<0.01	418 (4.7)	1000 (21)
Multiple times per day	0	2 (1.6)	0.16	90 (1)	168(3.5)
I have enough protected time for reading guidelines and scientific literature					
(Strongly) agree	17 (13.9)^a^	24 (19.5)^b^	0.24	1647 (34.2)	1600 (33.2)

Missing: ^a^8, ^b^7

### Change in the use of alternatives to face-to-face consultations

The number of Norwegian GP practices reporting daily use of digital text-based consultations on secure digital platforms increased from 41 (32%) to 88 (69.3%) (*p*<0.001), video consultations from 3 (2.4%) to 43 (33.6%) (*p*<0.001), and telephone consultations from 33 (25.9%) to 118 (92.2%) (*p*<0.001). In the international cohort, only numbers regarding the use of video consultations were available. The daily use of such consultations increased from 2.1% to 15.3%. See Table 3 for details.

**Table 3:** Change in prevalence of alternative consultation forms during the COVID-19 pandemic

	**Before COVID-19**^a^ **N (%)**	**Since COVID-19**^a^ **N (%)**	***P* value**
To what extent does this practice use digital consultations via secure digital platforms?			
Never	56 (43,8)	20 (15,7)	<0.001
Weekly or less	31 (24.2)	19 ( (15)	0.06
Daily or several times per day	41 (32)	88 (69.3)	<0.001
To what extent does this practice use telephone consultations?			
Never	40 (31,5)	0	<0.001
Weekly or less	54 (42.6)	10 (7.9)	<0.001
Daily or several times per day	33 (25.9)	118 (92.2)	<0.001
To what extent does this practice use video consultations?			
Never	102 (80.3)	13 (10.2)	<0.001
Weekly or less	22 (17.3)	72 (56.2)	<0.001
Daily or several times per day	3 (2.4)	43 (33.6)	<0.001
To what extent does this practice use video consultations? Total Pricov-19 cohort			
Never	4342 (85.4)	2657 (52.5)	
Weekly or less	639 (12.6)	1632 (32.2)	
Daily or several times per day	106 (2.1)	776 (15.3)	

Missing: ^a^2-3

### Change in work tasks, routines and governmental support

Survey items regarding change in GP tasks during the pandemic showed that 75 respondents (59.5%) experienced increased responsibilities in practice since the pandemic, and 49 (41.2%) indicated that they were happy with the task shift. In the international cohort, 80% reported an increased responsibility, and 28.6% were happy with the task shift. Seventy-nine percent of the Norwegian respondents reported that staff members were more involved in giving information to patients by phone than they were before. Among our participants, 32 (27.4%) felt that they received adequate support from the government, and 24 (20%) felt that guidelines from the government posed a threat to the well-being of the practice staff. The corresponding percentages in the international cohort were 24.2% and 33.1%. We found no significant associations between the reported task shifts and the rurality of the GP practice or the population size of the GP practice. For further details regarding practice changes since the pandemic, see Table 4.

**Table 4:** Reported practice changes since the COVID-19 pandemic (percentages that have indicated that they agree or strongly agree to the indicated statements). No associations with rurality or number of inhabitants in the municipality

**Survey items on task shifting**	**Practices who (strongly) agree**			
	**Norwegian cohort**		**Total Pricov-19 cohort**	
	**N**	**%**	**N**	**%**
Staff members are more involved in giving information/recommendations to patients by phone^a^	98	79	3772	81.8
Staff members are more involved in actively reaching out to patients that might postpone healthcare^b^	57	45.6	2608	57.5
Staff members are more involved in patient triage (by phone or in practice)^c^	95	74.8	3714	80.5
GPs are more involved in actively reaching out to patients that may postpone healthcare^c^	76	59.8	2900	59.3
My responsibilities in this practice increased^d^	75	59.5	3442	78.7
I am happy with the task shifting in my professional role since the COVID-19 pandemic^e^	49	41.2	1226	28.6
I do not feel prepared for the task shifting in my professional role^f^	14	11.4	989	23
Guidelines from government pose a threat to the good organization of this practice^g^	13	10.8	1341	28.7
Guidelines from government pose a threat to the well-being of the practice staff^g^	24	20	1549	33.1
Adequate support is provided by the government for the proper functioning of this practice^h^	32	27.1	1142	24.2

Missing: ^a^6, ^b^5, ^c^3, ^d^4, ^e^11, ^f^7, ^g^10, ^h^12

### Change in infection prevention measures

Patient triage by phone before appointments in the Norwegian practices increased from 67 (55.8%) to 113 (94.2%, *p*<0.001) after the start of the pandemic, and the number of practices that triaged the patients at the entrance increased from 13 (10.8%) to 55 (45.8%, *p*<0.001). The corresponding percentage after the pandemic in the international cohort was 73.5. The number of practices that always offered hand sanitizer to patients increased from 73 (58.4%) to 117 (95.1%, *p*<0.001). For further information on the change in infection prevention measures, see Table 5.

**Table 5:** Infection prevention measures before and after the pandemic

	**Norwegian cohort**			**Total Pricov-19 cohort**	
	**Before COVID-19**^a^ **N (%)**	**Since COVID-19**^a^ **N (%)**	***P* value**	**Before COVID-19** **N (%)**	**Since COVID-19**^a^ **N (%)**
Some staff members were a ring or a bracelet					
Never	11 (8.9)^a^	52 (42.3)^b^	<0.001	792 (16.1)	1568 (32)
Sometimes	63 (50.8)	51 ( 41.5)	0.14	2222 (45.1)	2253 (46)
Always	50 (40.3)	20 (16.3)	<0.001	1912 (38.8)	1081 (22.1)
The cleaning personnel uses a detailed protocol					
Never	22 (18)^c^	13 (10.7)^d^	0.11	845 (17.2)	525 (10.7)
Sometimes	21 (17.2)	16 (13.2)	0.38	1334 (27.2)	810 (16.5)
Always	79 (64.8)	92 (76)	0.05	2728 (55.6)	3575 (72.8)
Each GP consultation room is equipped with hand sanitizer					
Never	14 (11.3)^a^	4 (3.3)^b^	0.02	586 (11.8)	273 (5.5)
Sometimes	21 (16.9)	2 (1.6)	<0.001	678 (13.7)	97 (2)
Always	89 (71.8)	117 (95.1)	<0.001	3698 (74.5)	4575 (92.5)
Hand sanitizer is provided for patients at the door/ in waiting room					
Never	73 (58.4)^e^	5 (4.1)^b^	<0.001	2390 (48.3)	362 (7.4)
Sometimes	15 (12)	1 (0.8)	<0.001	837 (16.9)	203 (4.1)
Always	37 (29.6)	117 (95.1)	<0.001	1719 (34.8	4357 (88.5)
Patients are triaged by the door before entering practice					
Yes	13 (10.8)^f^	55 (45.8)^f^	<0.001	No data	3592 (73.5)
Limited amount of patients in waiting room					
Yes	5 (4.2)^f^	118 (98.3)^f^	<0.001	No data	4159 (85.1)
No use of the waiting room					
Yes	0^f^	9 (7.5)^f^	<0.01	No data	641 (13.1)
Increased amount of cleaning- and disinfection procedures					
Yes	6 (5)^f^	109 (90.8)^f^	<0.001	No data	3745 (76.6)
Structural measures in reception for the protection of staff					
Yes	22 (18.3)^f^	78 (65)^f^	<0.001	No data	2492 (51)
Patient triage per phone for the protection of staff					
Yes	67 (55.8)^f^	113 (94.2)^f^	<0.001	No data	3832 (78.4)

Missing: ^a^6, ^b^7, ^c^8, ^d^9, ^e^5, ^f^10

## Discussion

We found that during the COVID-19 pandemic, Norwegian GPs significantly increased their use of all forms of digital consultations, and there was an increased implementation of infection prevention measures and a noticeable task shift for both staff members and GPs.

### Alternatives to face-to-face consultations

We found a significant increase in the use of alternative consultations forms, both for digital text-based consultations, and telephone- and video consultations. This was as expected due to the need to reduce in-person consultations to reduce the risk of infection. Less restrictive rules for payment for telephone consultations introduced at the start of the pandemic also affected the use of alternative consultation forms. In addition, sick certificates before the pandemic could only be issued in face-to-face consultations, but this restriction was removed as an infection prevention measure.

Even prior to the pandemic, Norwegian patients were positive towards using various digital health services in contact with their GP [8]. Already before COVID-19, 32% of our GP respondents used digital text-based consultations on a daily basis, hence it was probably easy for both patients and GPs in many practices to increase this means of consultation when the government recommended as few face-to-face contacts as possible. This is mirrored in the increase of up to almost 70% in daily use of digital text-based consultations since the pandemic.

The significant change in the use of alternatives to face-to-face consultations also echoes the findings of the Norwegian Corona Commission, both for GP practices and the specialist health care system [1]. Such a change appeared to happen in parallel to a change in attitude of GPs. In a study looking at GPs’ experiences with video consultations early in the pandemic, including 1237 Norwegian regular GPs during April and May 2020, more than half of the GPs responded that they consider video consultations as good as or even better than in-person consultations for previously known patients [9]. Moreover, they estimated that around 20 percent of the consultations in a normal non-pandemic situation could be performed digitally. They considered the pandemic situation as a learning situation that could potentially improve the implementation of digital solutions, discovering in which situations digital solutions can be beneficial or not.

An increase in video consultations was also found in the international PRICOV-19 cohort, where the indicated daily use of video consultations increased from 1.3 to 10.5 % [10]. It is possible that Norwegian GP practices more easily than many other countries could launch such services, as all Norwegian GPs already used electronic patient record systems with a mandatory connection to a high-security digital platform. This may explain the larger increase in the Norwegian cohort. An interesting topic for further research will be to investigate whether GPs and patients will continue their new habits in the use of digital consultations when infection prevention is no longer on top of the agenda.

### Change in work tasks and routines

Almost 60% of the Norwegian respondents answered that their practice responsibilities increased during the pandemic. In the total PRICOV-19 data, this percentage was even higher at 77.6 % [11], possibly reflecting that Norwegian GPs largely run their own practices and as such already have a high degree of responsibility in practice. Of the Norwegian GPs, 41.2% were happy with the experienced task shift during the pandemic. Furthermore, in an international comparison between the countries in the PRICOV-19 study, Norway was among the countries who were most happy with the task shift [12]. Among our participants, only 11% felt they were not prepared for this shift in their professional tasks, and in international comparisons Norway was among the countries with the lowest proportion that indicated a lack of preparedness. This may reflect that Norwegian general practice is used to offering a wide range of services [5], and also that Norwegian GPs have high expectations for themselves to offer comprehensive services [13].

The first weeks after the lockdown of the Norwegian society in March 2020 there was a reduction in the normal patient flow, and the GPs worried that chronically ill patients could postpone necessary health services in fear of infection [14]. Our results show that this prompted a more active outreach to vulnerable patients, as both staff members and GPs indicated an increased involvement in reaching out to such patients. The Norwegian system, where each GP has a designated patient population, facilitates such outreach activity.

### Increased work pressure and available time for professional update

Statistics from the Norwegian Directorate of Health demonstrate that the number of patients in contact with their GP was higher in March 2020 than in the same month in 2019. For the remainder of 2020, the contact rate was the same as or higher than in 2019. An increased share of contacts were digital or by phone. Contacts due to respiratory symptoms increased from the onset of the pandemic, and significantly so during March and April 2020, most likely due to uncertainties regarding symptoms of COVID-19. Another notable development was that the number of consultations due to mental health problems increased in November and December 2020 compared to 2019 [1]. This added to the extensive tasks of GPs, like daily briefing meetings, following up on new guidelines, performing vaccination, prioritization according to risk, and clinical follow-up of confirmed infected patients, posing an extra strain on Norwegian GP practices that already experienced work-overload in a normal situation before the pandemic [15].

A relatively low percentage of Norwegian GPs indicated that they had enough time for professional/scientific updates both before and after the pandemic. The international PRICOV-19 cohort reported more protected time for update than the Norwegian GPs. This may point to Norwegian general practice having a high focus on clinical tasks, possibly due to most GPs being self-employed and with a high proportion of fee-for-service income. There is also a documented high workload among Norwegian GPs [15, 16]. There is an ongoing process in Norway to alleviate the work pressure of and improve the recruitment to general practice [17], and the need for protected time for professional update should be addressed in this process.

### Support from the government

Only about one in four of our Norwegian respondents felt that they received adequate support from the government, this is comparable to the result for the total PRICOV-19 data [11]. In a Norwegian qualitative study, several GPs reported that they felt a lack of support from their Municipality Chief Medical Officer (MCMO), that it was difficult to know who in the municipality to contact and that it was difficult to navigate the guidelines [14]. In contrast, they reported that the Norwegian GP Association and the GP College were very valuable sources of information and support through the pandemic. The low percentage who felt they received enough support should be a heads-up for Norwegian health authorities. Norwegian GPs, although mostly self-employed, are contracted with the municipality and are expected to follow official guidelines and advice. Hence, a good strategy for communication and support between the health authorities and the GPs should be established to prepare for future health incidents similar to the COVID-19 pandemic.

In the complete PRICOV-19 data, 28.7% indicated that governmental guidelines were perceived to pose a threat to the organization of the GP practice, as opposed to only 10.8% of Norwegian respondents. Despite the lack of governmental support, it seems that Norwegian GPs are relatively content with the guidelines provided.

### Change in infection prevention measures

A European qualitative study found that GPs felt uncertainty regarding how they should follow all guidelines on infection control, as well as whether they would receive financial coverage for the increased costs related to infection prevention measures [18]. This may have led to both an unwanted variation in such measures, as well as a reluctance to implement these measures for financial reasons. However, in a previous study analysing data from the total PRICOV-19 data, all examined infection prevention measures increased significantly compared with the situation before the pandemic [19], and this is in line with our results. A better support from the authorities, as discussed above, could have alleviated uncertainty and reduced unwanted variation.

More respondents in the total international data than in the Norwegian cohort reported that they triaged patients at the door, but more Norwegian respondents reported patient triage per phone. This may be due to differences in practice organization between countries.

### Strengths and limitations

The study used a validated survey. Our results provide knowledge on the primary health care organisation during the COVID-19 pandemic, where there is still a paucity of data [20, 21]. A further strength of this Norwegian study is that data from the whole country were available, which is important due to large geographical variation in the country.

However, several limitations should be noted. There was a possible selection bias as participation in this study was voluntary. The invitation to participate in the study went to all Norwegian GPs, and it is possible that the respondents were particularly interested in the topic. The response rate for Norway was lower than the median value of 22.0% among the international cohort. Also, the study is cross-sectional, meaning it doesn´t tell us how the study topics may have changed during the pandemic. In addition, a few outcome variables focused on differences between the situation before and since COVID-19. These results should be interpreted carefully as practices that already performed well could not make the same progress as other practices.

## Conclusion

We found a significant change in several aspects of Norwegian general practice and in particular an increase in the use of alternatives to face-to-face consultations. GPs and other staff members were more involved in reaching out to vulnerable patients after the onset of the pandemic. Compared to the total PRICOV-19 cohort, Norwegian respondents were more content with the task shift that happened due to the pandemic. Only one in four GPs felt that the support from the government was adequate, an important point for further consideration by the health authorities.

The results of this study are valuable to future preparedness plans and may help secure high-quality care in general practice in Norway during future periods with a high prevalence of patients with possible communicable diseases.

## Data Availability

All data are centrally stored on the server of Ghent University (Belgium). All data was anonymized at Ghent University, and all raw data that could lead to the identification of the respondents was permanently removed. Reasonable request is required to access non-identifiable data by users who are external to the PRICOV-19 consortium. Access will be subject to a data transfer agreement and following approval from the principal investigator of the PRICOV-19 study.

## Abbreviations

GP: General practitioner
MCMO: Municipality Chief Medical Officer
PRICOV-19: The research project “Quality and Safety in Primary Care in times of COVID-19

## References

[1] Norwegian Corona Commission. The authorities´ handling of the COVID-19 pandemic. Report part I. NOU 2021:6. Oslo. 2021. https://www.regjeringen.no/contentassets/5d388acc92064389b2a4e1a449c5865e/no/pdfs/nou202120210006000dddpdfs.pdf.

[2] Norwegian Corona Commission. The authorities´ handling of the COVID-19 pandemic. Report part II. NOU 2022:5. Oslo. 2022. https://www.regjeringen.no/contentassets/d0b61f6e1d1b40d1bb92ff9d9b60793d/no/pdfs/nou202220220005000dddpdfs.pdf.

[3] Comas-Herrera A, Patel D, Arling G, Mossong J, Schmidt A. International data on deaths attributed to COVID-19 among people living in care homes. 2022. Available from: https://ltccovid.org/2022/02/22/international-data-on-deaths-attributed-to-covid-19-among-people-living-in-care-homes/.

[4] Matenge S, Sturgiss E, Desborough J, Hall Dykgraaf S, Dut G, Kidd M (2022). Ensuring the continuation of routine primary care during the COVID-19 pandemic: a review of the international literature. Fam Pract..

[5] Eide TB, Straand J, Björkelund C, Kosunen E, Thorgeirsson O, Vedsted P (2017). Differences in medical services in Nordic general practice: a comparative survey from the QUALICOPC study. Scan J Prim Health Care.

[6] Van Poel E, Vanden Bussche P, Klemenc-Ketis Z, Willems S (2022). How did general practices organize care during the COVID-19 pandemic: the protocol of the cross-sectional PRICOV-19 study in 38 countries. BMC Prim Care.

[7] Harris PA, Taylor R, Minor BL, Elliott V, Fernandez M, O'Neal L (2019). The REDCap consortium: Building an international community of software platform partners. J Biomed Inform..

[8] Zanaboni P, Fagerlund AJ (2020). Patients’ use and experiences with e-consultation and other digital health services with their general practitioner in Norway: results from an online survey. BMJ Open.

[9] Johnsen TM, Norberg BL, Kristiansen E, Zanaboni P, Austad B, Krogh FH (2021). Suitability of Video Consultations During the COVID-19 Pandemic Lockdown: Cross-sectional Survey Among Norwegian General Practitioners. J Med Internet Res.

[10] Adler L, Vinker S, Heymann AD, Van Poel E, Willems S, Zacay G (2022). The effect of the COVID-19 pandemic on primary care physicians in Israel, with comparison to an international cohort: a cross-sectional study. Isr J Health Policy Res.

[11] Collins C, Clays E, Van Poel E, Cholewa J, Tripkovic K, Nessler K (2022). distress and wellbeing among general practitioners in 33 countries during COVID-19: results from the cross-sectional PRICOV-19 study to inform health system interventions. Int J Environ Res Public Health.

[12] Groenewegen P, Van Poel E, Spreeuwenberg P, Batenburg R, Mallen C, Murauskiene L (2022). Has the COVID-19 Pandemic Led to Changes in the Tasks of the Primary Care Workforce? An International Survey among General Practices in 38 Countries (PRICOV-19). Int J Environ Res Public Health.

[13] Eide TB, Straand J, Rosvold EO. Patients’ and GPs’ expectations regarding healthcare-seeking behaviour: a Norwegian comparative study. BJGP Open. 2018. 10.3399/bjgpopen18X101615. 10.3399/bjgpopen18X101615 PMC6348319 30723801

[14] Heltveit-Olsen SR, Lunde L, Brænd AM, Spehar I, Høye S, Skoglund I, et al. Experiences and management strategies of Norwegian GPs during the COVID-19 pandemic: a longitudinal interview study. Scand J Prim Health Care. 2023;41(1):2–12. 10.1080/02813432.2022.2142796 PMC10088916 36350846

[15] Svedahl ER, Pape K, Toch-Marquardt M, Skarshaug LJ, Kaspersen SL, Bjorngaard JH (2019). Increasing workload in Norwegian general practice - a qualitative study. BMC Fam Pract.

[16] Morken T, Rebnord IK, Maartmann-Moe K, Hunskaar S (2019). Workload in Norwegian general practice 2018 - an observational study. BMC Health Serv Res.

[17] Kjetil Telle et al. Evaluation of the general practcie services. First report of the expert group. 2022. Available from: https://www.regjeringen.no/contentassets/260a4d50ceff4195a13090c2faf92ea9/ekspertutvalgets-forelopige-rapport-1.-desember.pdf2022.

[18] Wanat M, Hoste M, Gobat N, Anastasaki M, Böhmer F, Chlabicz S (2021). Transformation of primary care during the COVID-19 pandemic: experiences of healthcare professionals in eight European countries. Br J Gen Pract.

[19] Collins C, Van Poel E, Šantrić Milićević M, Tripkovic K, Adler L, Bjerve Eide T (2022). Practice and system factors impact on infection prevention and control in general practice during COVID-19 across 33 countries: results of the PRICOV cross-sectional survey. Int J Environ Res Public Health.

[20] Kurotschka PK, Serafini A, Demontis M, Serafini A, Mereu A, Moro MF (2021). General Practitioners' Experiences During the First Phase of the COVID-19 Pandemic in Italy: A Critical Incident Technique Study. Front Public Health.

[21] Windak A, Frese T, Hummers E, Klemenc Ketis Z, Tsukagoshi S, Vilaseca J (2020). Academic general practice/family medicine in times of COVID-19 - Perspective of WONCA Europe. Eur J Gen Pract..

